# The Effect of Women’s Empowerment in the Utilisation of Family Planning in Western Ethiopia: A Structural Equation Modelling Approach

**DOI:** 10.3390/ijerph18126550

**Published:** 2021-06-18

**Authors:** Muluken Dessalegn Muluneh, Lyn Francis, Mhiret Ayele, Sintayehu Abebe, Misrak Makonnen, Virginia Stulz

**Affiliations:** 1 Amref Health Africa in Ethiopia, P.O. Box 20855, Addis Ababa 1000, Ethiopia; Mihret.Ayele@Amref.org (M.A.); Sintayehu.Abebe@Amref.org (S.A.); Misrak.Makonnen@Amref.org (M.M.); 2 School of Nursing and Midwifery, Parramatta South Campus, Western Sydney University, Parramatta, NSW 2151, Australia; L.Francis@westernsydney.edu.au; 3 School of Nursing and Midwifery, Western Sydney University, Locked Bag 1797, Penrith, NSW 2751, Australia; v.stulz@westernsydney.edu.au

**Keywords:** women’s empowerment, family planning, decision making

## Abstract

This study examines the associations between women’s empowerment and family planning use in Jimma Zone, Western Ethiopia. A total of 746 randomly selected married women of reproductive age were interviewed. The data were employed by structural equation modelling (SEM) to investigate the complex and multidimensional pathways to show women’s empowerment domains in family planning utilisation. Results of the study revealed that 72% of married women had used family planning. Younger women, having access to information, having access to health facilities and being aware about family planning methods, living in a rural area, having an older partner and increased household decision-making power were associated with using family planning methods. Women’s empowerment is an important determinant of contraceptive use. Women’s empowerment dimensions included increased household decision-making power, socio-demographic variables and having access to information about family planning and accessible health facilities. These were found to be important determinants of contraceptive use. Future interventions should focus on integrating women’s empowerment into family planning programming, particularly in enhancing women’s autonomy in decision making. Further research is warranted on the socio-cultural context of women that influences women’s empowerment and family planning use to establish an in-depth understanding and equity of women in society.

## 1. Background

Globally, approximately over 290,000 maternal deaths every year and about 99% of the global maternal mortality rates occur in developing countries, with the Sub-Saharan Africa (SSA) region reporting two-thirds (66%) of maternal deaths [1]. Evidence has conveyed that women’s health is critical for the health of the entire family and health service utilisation is paramount [1]. However, little attention has been focused on women’s empowerment in the utilisation of family planning in SSA [1].

The fifth Sustainable Development Goal (SDG) is to achieve gender equality and empowerment of all women and girls [2]. Reducing gender inequality is a vital policy agenda globally and access and rights to resources are essential for women’s health. The effects of gender inequities persist throughout all phases of women’s lives and play a role in determining years of education, opportunities for career and employment, age at marriage and number and timing of children [3,4]. Consequently, gender inequities compromise the rights, safety, well-being and health of women and girls. Gender inequity affects sexual and reproductive health outcomes including unintended and teenage pregnancy, an increased incidence of human immunodeficiency virus (HIV), increased rates of violence against women and girls and increased vulnerability to other sexual risk behaviours [1,4,5,6]. Women’s inequality in accessing various services is a public health challenge and a human rights violation, and reverting this is critical to achieving SDG targets, including health [3,5,7]. Women’s increased political participation, control of resources including land, access to employment and education are crucial for promoting sustainable development [8].

Greater gender equality can lead to improvements in health and quality of life for women and their family members by numerous pathways [4,9,10]. Evidence suggests that gender equality or women’s empowerment positively influences family planning use [1,3]. Gender inequities limit women’s access to good-quality family planning services. They also hinder women’s ability to negotiate family planning and use contraception effectively. In addition, traditional gender roles generally place greater barriers to women’s access to family planning services [1,4,11,12].

The implicit assumption is that in most societies, particularly in SSA including Ethiopia, men control the women of their social class, especially in their households, families and health service utilisation including use of family planning [2,3,13]. At the same time, improved health outcomes for women can help to strengthen their empowerment [1,14]. Healthy women are more able to actively participate in society and improve economic development by participating in their own social and economic interests [1,14,15]. They are also more likely to have greater bargaining power and control over resources within the household [1,14,16].

Maternal, neonatal and child health (MNCH) specifically focuses on women’s health and children and infants under the age of five. Family planning contributes to women’s health by ensuring women are educated about fertility and contraceptive use so that they can make decisions about their family size [11]. There has been increased investment towards family planning for women’s health. However, the unmet needs for family planning have declined slowly in many Sub-Saharan African (SSA) countries including Ethiopia [17]. Investing in family planning is one of the smart investments for development as population dynamics have a fundamental influence on the pillars of sustainable development [18,19]. Most importantly, the use of modern contraceptives is known to be highly cost-effective and has poverty-reducing effects, as demonstrated in earlier studies [18,20,21]. Modern contraceptives also assist women in their personal decisions about family size [11]. Despite family planning’s benefits to women’s health, changes in maternal and child health for women in Ethiopia are limited [22], however, over the last two decades, Ethiopia has made progress in the areas of maternal, neonatal and child health (MNCH) including reproductive health [19,23,24].

Notable achievements in maternal and child health include a 67% drop in the under-five mortality rate from 1990 estimates, which contributed to an increase in average life expectancy from 45 to 64 years in 2016; the maternal mortality ratio being reduced by 71% to 420/100,000 live births; and the contraceptive prevalence rate being increased from 3% to 36%, leading to a reduction in the total fertility rate from 7.7 in the 1990s to 4.1 in 2014. Modern contraceptive use for currently married women has steadily increased over the last 16 years in Ethiopia from 6% in 2000 to 35% in 2016 [19,23,24]. Regardless, the progress of contraceptive use remains slow [19,24]. Contraceptive use is not uniform across regions and population groups and particularly in Ethiopia [19,24].

### Research Hypothesis

The concept of women’s empowerment is complex, as there is considerable variation in its conceptualisation. Most definitions link empowerment with the power or freedom used to achieve desired outcomes [25]. The World Bank goes beyond this and defines empowerment as the “expansion of freedom of choice and action to shape one’s life” [26]. Kabeer (1999) defines women’s empowerment as a “process by which those who have been denied the ability to make strategic life choices acquire such an ability.” [1] Among many frameworks proposed to measure empowerment, the framework proposed by Maholtra, Schuler and Boender (2002) is amongst one of the most comprehensive, in which women’s empowerment is measured in five dimensions and at different levels [27]. The framework suggests that women’s empowerment could be exercised in five different arenas: household economy, socio-cultural activities, legal activities, politics and psychology.

**Figure 1:**
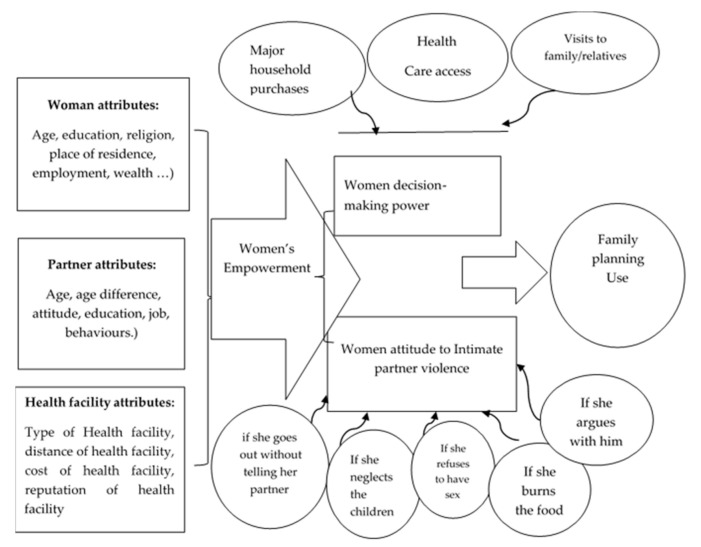
Conceptual framework.

The other framework used by Haque et al. (2011) [28] in Bangladesh consisted of three key dimensions: economic decision making, household decision making and women’s physical mobility. In this framework, women are classified as participating in decision making if they make a decision alone or jointly with their partner. The dynamic and multidimensional nature of women’s empowerment, and its existence at various levels, makes it challenging for researchers to measure empowerment. An attempt was made to assess the overall effect of women’s empowerment and its various dimensions on contraceptive use by adapting the structured framework developed by the DHS program as a standard measurement [29]. Women’s empowerment measures [24] are demonstrated in Figure 1. As shown in Figure 1, women’s attributes, partner attributes and health facility access might positively affect multiple dimensions of empowerment, which in turn promotes family planning use. Women’s empowerment is hypothesised as a predictor of reproductive health outcomes. It is believed that empowered women are more likely to plan their pregnancies, delay marriage, receive prenatal care and visit a skilled health provider during pregnancy and childbirth. It is hypothesised that empowered women are more likely to use contraception in comparison to women who are not (or are less) empowered.

## 2. Methodology

### 2.1. Study Design, Population and Setting

A community-based cross-sectional study was employed to collect relevant data for the study. The study population included all women of the reproductive age group (15–49 years) who were married or living together with a partner and lived in the study area for at least six months preceding the survey.

The study was conducted in selected districts of Jimma Zone, which is located 352 km from the capital Addis Ababa in the southwest of Ethiopia. The zone consists of 20 rural districts and one special town administration (Agaro Town), and 46 small urban and 512 rural kebeles (smallest formal administrative units). The study focused on predominantly Muslim communities [24].

### 2.2. Sample and Sampling Procedure

Sample size was calculated using the OpenEPI single proportion formula. A total sample size of 746 respondents was included considering the following assumptions: contraceptive prevalence (63.5%) rate of Ethiopia amongst married women [22], 5% margin of error (d), design effect (DE) of 2 and 5% non-response rate.

Multistage cluster sampling techniques were employed, where districts and kebeles were considered as the primary and secondary sampling units, and households were selected randomly from the selected district. A total of four districts and eight kebeles were selected randomly. The total sample size was proportionally allocated to each selected kebele based on the number of households in each kebele.

### 2.3. Data Collection and Measurement Tools

Primary data were collected using a structured questionnaire using mobile applications, ODK/KOBO, where a pretested structured questionnaire with pre-coded answers was uploaded. The questionnaire was adopted and developed from EDHS 2016 [24], women’s empowerment scale and related literature considering the local context and the nature of the project intervention. Data collection tools were translated into the local language and then translated back to English to check their consistency (Supplementary File S1). The data are freely available (Supplementary File S2).

### 2.4. Data Analysis

The analysis followed a structural equation model (SEM). Note that the SEM technique is the combination of factor analysis and multiple regression analysis and it is used to analyse the structural relationship between measured variables and latent constructs. The authors preferred this analysis as it is more powerful than regression analyses and it examines linear causal relationships amongst variables, whilst simultaneously accounting for measurement error. Our SEM has two latent constructs, including individual empowerment indicators, to represent each empowerment dimension. The latent variable SEM included family planning use, decision-making power and attitude towards violence as three “endogenous variables” and 12 “exogenous variables”.

*Three endogenous variables: Family planning* was measured as ever using contraceptives amongst women who were married or had a male partner. Based on women’s responses, it was coded as a binary response of yes if they did, or no if they never used family planning methods. For example, the following question was asked: Have you ever done something or used any modern contraceptive method to delay or avoid getting pregnant? *Household decision-making power* was examined as a latent construct consisting of three indicators: women’s participation in decisions regarding their own health care, major household purchases and visits to family/relatives. These questions were asked only to women who were currently married/had a partner. The variables were first recoded to examine if women participated in decisions (i.e., alone/jointly with their husband/partner) or not. A latent variable was constructed from the three binary variables for each decision. *Attitudes towards violence* were examined as a latent construct consisting of five indicators concerning a woman’s acceptance of a wife’s physical violence by her husband/partner. The survey asked about the following five situations: if she goes out without telling him, neglects the children, refuses to have sex with him, argues with him or burns the food. Each variable was first recoded into binary data (i.e., yes accept, or no).

*Twelve exogenous variables:* Exogenous variables included: current age of the woman, employment, access to information, access to health facilities, awareness about contraceptives, husband’s/partner’s employment status, source of income for the household, woman’s and her partner educational status, wealth status of the family, residing in an urban or rural area, religion, if the woman faced any form of violence in the past and if she and her partner chewed khat or consumed alcohol. Access to health facilities is multidimensional and the following question was asked: Do you have access to a health facility nearby (health center/health posts)? Each response was recorded either as yes or no as binary data. We also asked questions about distances in kilometres travelled, availability of commodities, quality and satisfaction and cost implications for related health access. In Ethiopia, contraceptive use at public health facilities is free of charge and covered by the government.

### 2.5. Operational Definitions

Women’s empowerment refers to women who participate in all decision making about major purchases, health care access and visits to family and relatives.

The decision-making skill of women refers to women participating in decision making if they make decisions alone or jointly with their partner.

Gender-based violence refers to any public or private act of sexual and gender-based violence that results in, or is likely to result in, physical, sexual or psychological harm or suffering to women, including threats of such acts, coercion or arbitrary deprivation of liberty [3].

Access to health facilities refers to nearby access to health facilities from their living residence/community.

Contraceptive use refers to a modern method or device used to prevent pregnancy. Traditional methods of birth control were not included.

Contraceptive awareness refers to being informed about modern contraceptives’ methods, purpose and use.

## 3. Results

### 3.1. Background Characteristics of the Study Respondents

Data for the research were collected from a total of 746 women of reproductive age (15–49 years) who were married or had a partner. The mean age of the respondents was 29.9 (standard deviation (SD) ± 7.42) years, and the minimum and maximum ages were 16 and 49, respectively. By religious affiliation, the vast majority (97%) of the respondents were Muslims, almost three-quarters of women (74%) worked in agriculture (land cultivation (60.86%) and animal husbandry (13.67%) and only a minority (3.49%) of the respondents work in skilled work (see Table 1). Almost two-thirds (62.87%) of the respondents in the study area had no access to external communication information (TV/radio/magazine).

The study indicated that women were relatively younger (about ten years) in comparison to their husband/partner with the average age of the partner being 38.29 (SD ± 9.48) years (t = −19.059, *p* = 0.000). Similar trends were observed for the spousal gap in schooling. Women were less educated in comparison to their husbands/partners. More than half (51.74%) of the women respondents were illiterate while a relatively smaller proportion of their partners (40%) were illiterate. Women in the study area were relatively less literate than men (see Table 1).

### 3.2. Age at First Marriage and Birth

Results of the study show that the first marriage amongst women aged 15–49 occurred by age 17.71 years on average and the median age at first marriage was 18 years, which is higher than the national figure (17.1) [24]. Age at first marriage has a major effect on childbearing because women who marry early have on average a longer period of exposure to the risk of pregnancy and give birth to a greater number of children over their lifetime.

The vast majority of the respondents (96.92%) have birthed and have four children on average, of which about four are alive (see Table 2 below). Almost half of the respondents (46%) reported that the birth interval between the youngest and older child was reported to be less than two years, which is considered a short interval [24]. Short intervals, particularly if less than two years, are strongly associated with childhood mortality and increased risks for other health problems [24]. A considerable proportion of the respondents (15%) reported that their husband/partner has another partner (see Table 2).

### 3.3. Family Planning Utilisation

Our study revealed that 72% of married women aged between 15 to 49 years utilised family planning services during the course of their lifetime. It was also found that more than half (55%) of the respondents were current users of family planning services.

### 3.4. Participation in Major Household Decisions

Results of the study revealed that more than two-thirds (68%) of the study participant women reported that they are involved in decisions regarding large household purchases either alone or jointly with their husband/partner, whereas almost a quarter (24%) of the respondents reported that their husband or someone else makes the decision to visit their family or relatives. Likewise, less than a fifth (19.84%) of the respondents reported that their husband or partner makes the decisions regarding women’s health care (see Table 3 below). According to the EDHS (2016) [24], women are considered to participate in household decisions if they make decisions alone or jointly with their partner/husband in all the three dimensions presented above. In this regard, the results of the study imply that women’s participation in household decisions in the study area was limited, particularly in social interaction and accessing health care.

### 3.5. Attitudes towards Violence

A half to two-thirds of the sample believed that a husband is justified in physical violence towards his partner/wife in the first three key dimensions considered in this study (for dimensions 1 to 3, see Table 4 below). Almost half of the sample thought physical violence was justified “if they burnt food and if they refused to have sex” (for dimensions 4 and 5, see Table 4).

### 3.6. Factor Analysis (CFA) Results

Factor analysis was employed to identify the underlying structure of the observable data from the survey responses. Therefore, the correlation coefficients between the factor and the original variables (factor loadings) were used to understand the structure of latent factors in the model. After running the analysis, the results revealed two dimensions. The first dimension, household decision-making power, includes decisions about large household purchases, participation in public meetings and decisions about the health care of women.

The second factor of women’s empowerment found that women’s attitude towards violence comprised five dimensions including physical violence, which were going out without telling her husband/partner, neglecting the children, arguing with him, refusing to have sexual intercourse with her husband/partner and burning the food.

Table 5 presents the EFA results that indicate two factors (eigenvalues > 1.0) [30]. Household decision-making power (three indicators) and attitudes towards violence (five indicators) were tested. The two factors were significantly, yet not highly, correlated with factor correlations of less than 0.31 (*p* < 0.05), indicating the distinction of each factor. As a rule of thumb, variables with factor loadings of 0.4 and above are very significant to determine the minimum loading necessary to comprise the items [31]. For instance, Table 5 shows that decisions about going to a public meeting is the highest factor loading for decision-making power and physical violence if the woman refuses sexual intercourse and if she neglects her children are the highest factor loadings for attitude towards physical violence. As a result, both factors have been selected. The factor scores obtained for each respondent on the two factors extracted were used as key independent variables to ensure the reliability of the results from the structural equation model (see Table 5).

### 3.7. Structural Equation Modelling Results (SEM) 

The final adjusted SEM result is presented in the table below (see Table 6). We used SEM as it is more powerful than regression analysis as it examines linear causal relationships amongst variables, whilst simultaneously accounting for measurement error. The standardised path coefficients and *p* values are reported. The model fit statistics (RMSEA, CFI and SRMR) show that the model fits the data well (RMSEA = 0.046, CFI = 0.91, SRMR = 0.036) (see Table 6).

**Figure 2:**
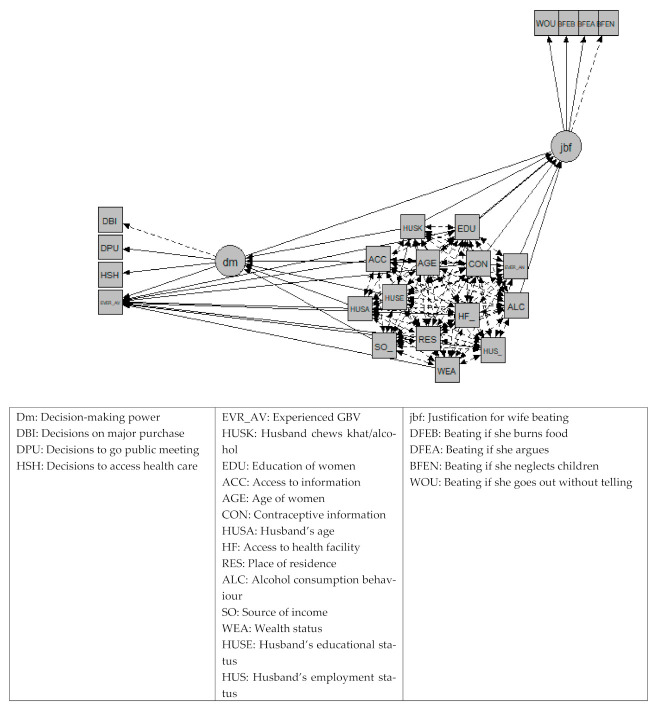
Structural equation model.

The woman’s increased decision-making power and her aversiveness towards violence were positively related to family planning use. In addition, other factors were significantly related to family planning use: having access to health facilities, having awareness about family planning and the partner being employed. Alternatively, the younger the partner, being from a middle or higher class and living in rural areas showed a negative relationship with family planning use. Other demographic characteristics were significantly but differently related to empowerment dimensions. Amongst the significant covariates, older age of the woman, having a higher income, the husband being literate and the husband not chewing khat and not consuming alcohol had a positive relationship with her increased decision-making power. If the partner was younger, more decision making occurred for the woman. Moreover, having no awareness of contraceptive use was associated with violence being more acceptable, whereas if the woman had ever attended school of any level, was older and the partner did not consume khat and alcohol, this led them to being less likely to accept violence (Figure 2).

Figure 2 shows a diagram of the latent variable SEM. *Ever_AV*: using family planning to avoid pregnancy, dm: decision-making power, *jbf*: positive attitude towards violence. Standardised path coefficients are reported with the following significance levels: *** *p* < 0.001, ** *p* < 0.01, * *p* < 0.05. Factor loadings and correlations among disturbances are all significant at *p* < 0.001.

## 4. Discussion

This study revealed that women’s empowerment is variable and affects their choice of and access to family planning. In this study, the analysis enabled comparisons and testing of several dimensions simultaneously about women’s family planning use. This study investigated the association between contraceptive use and various components of women’s empowerment (three dimensions of decision-making skills and five dimensions of attitudes to physical violence towards wives). The significant association between women’s empowerment and contraceptive use is consistent with the results from previous studies where women’s empowerment was positively associated with the use of health care services in 67 developing countries [32] and a study conducted on women’s empowerment as an enabling factor of contraceptive use in Sub-Saharan Africa [4,33]. Many individual studies in African countries found the same results, showing that that if women were more empowered, they were more likely to use modern contraception [4,10,12,15,34,35]. We propose to develop an integrated approach, to ensure that women’s empowerment and other attributes improve family planning, rather than proposing a standalone intervention.

The reference point for using family planning services amongst married women and unmarried women is different [36,37]. Regardless of their background status, unmarried women will use family planning services to avoid getting pregnant [15,19,24]. Additionally, the influence of a male partner is nonexistent amongst unmarried women [24]. For unmarried women, the influence of decision-making is also different in comparison to married women. Married women in the Ethiopian context are expected to bear children [15,24,35]. Therefore, the status of a woman’s empowerment (decision-making and violence) amongst married women and unmarried women can affect family planning utilisation preference differently [37]. The relationship between family planning utilisation preference and empowerment can be measured more effectively amongst married women in comparison to unmarried women [35,36,37].

Services being made available for women is not the absolute solution to improve family planning use as this is affected by many factors [18,38]. Rather, integrating women’s empowerment, such as improving women’s negotiation skills about the use of family planning services, and educating women about the available family planning services, would boost contraceptive use amongst women [11,21,38]. The empowerment of women is critical in family planning programming to enhance the overall improvement and utilisation of family planning services.

Women’s empowerment had a stronger effect in our analysis when we tested it independently against family planning utilisation. Increased power of making decisions on major household purchases, seeking health care services and attending public meetings were also positively influenced by the older age of the woman and her partner consuming less alcohol, less substance use behaviour and his educational status being higher. One of the most common reasons for not using contraceptives included lack of access to information and health facilities [39], and this may be attributed to limited access to supplies, in which case health facilities and health care providers have the potential to address and target these challenges. This limited access to health facilities is consistent with our study, resulting in women having limited access to contraceptives and, subsequently, utilisation rates are decreased. Therefore, addressing women’s empowerment could provide a multipronged boost in the utilisation of the services. This could also be related to the cost of transport and lack of understanding about the benefits of using contraceptives.

Similarly, we found that family planning utilisation amongst women living in rural areas was limited, which resonates with other studies [17,35]. This could be explained by women residing in rural areas being less educated and having a limited understanding about the significance of family planning. Moreover, rural health facilities may have limited contraceptive supplies for women due to a number of factors, including shortages of supplies, lack of trained health providers and other challenges in the health system [18].

Some studies have also found that wealthier women are more likely to use contraceptives [9,19,20,33]. In contrast, our study showed that wealthier women were less likely to use contraceptives. The significance of this finding is that this may be related to women who are wealthier wanting to have more children as they have the capacity to support the children financially without fearing their husband/partner. Additionally, wealthier women are more likely to have more children in the Muslim community [19,24,33]. Furthermore, our study showed that older women were less likely to use contraceptives, although there may be a tendency that wealthier women who are older are less likely to use contraceptives, which is consistent with other studies [11,21]. In contrast to our study, increased use of family planning methods was found with higher economic status.

### 4.1. Strengths and Limitations of the Study

The major strength of this study is the use of the standard measurement for women’s empowerment, which makes the findings of the study comparable with other studies that have used the DHS. Despite the authors employing SEM, this study has some limitations. Prominently, the analyses used cross-sectional data, hence, only associations and no causal relationships were established. The representativeness of the study sample and generalisability of the results are limited due to the omission of women who were unmarried. Additionally, some variability will occur depending upon the context, such as culture, urbanisation, immigration pattern and pastoral nature of the country. Furthermore, social desirability and recall bias could occur in studies that deal with lifetime contraceptive use.

### 4.2. Conclusion and Recommendations

Based on the findings of this study, it is imperative to note that enhancing women’s empowerment could assist in improving contraceptive utilisation. Women’s empowerment is multifactorial, especially in relation to improved decision making that plays a vital role in health quality outcomes of women. Thus, contraceptive utilisation is of paramount importance in the health of women and their children.

Moreover, this study highlights that not only are the demographic characteristics of a woman and her partner (age and wealth) significant indicators when considering violence but limited access to family planning health facilities also affects contraceptive use for women living in Ethiopia. Future interventions should focus on integrating women’s empowerment through various means such as improving education and economic status and improving negotiation skills for enhanced autonomous decision making regarding family planning use. Further research is warranted regarding the socio-cultural context of women and factors that influence women’s empowerment and family planning use to establish an in-depth understanding so that equitable norms in society are upheld.

## Figures and Tables

**Table 1:** Sociodemographic features of the respondents and their partners, Western Ethiopia, 2020.

Characteristics	Frequency	Percentage (%)
Educational status of women		
Illiterate	386	51.70
Primary (1–8)	276	37.00
Low secondary (9–10)	62	8.30
Secondary and above	22	3.00
Partner education status		
Illiterate	299	40.08
Primary (1–8)	355	47.59
Low secondary (9–10)	55	7.37
Secondary and above	37	4.96
Employment		
Not employed	701	93.97
Employed	45	6.03
Religious denomination		
Muslim	723	96.92
Christian	23	3.08
Main source of income for the family		
Land cultivation	454	60.86
Small business	137	18.36
Husbandry	102	13.67
Paid job	26	3.49
Labour	23	3.08
Other	4	0.53
Access to sources of information		
Yes	469	62.87
No	277	37.13

**Table 2:** Birth, age at first marriage and fertility among married women in Jimma Zone, Western Ethiopia, 2020.

Have You Ever Given Birth?		Frequency	Percentage
Yes		723	96.92
No		23	3.08
How many children do you have in your life (both alive and dead)?	Mean	Median	SD
	4.02	4.00	2.44
Total number of children alive	3.63	3.00	2.22
Birth interval		Frequency	Percentage
One year		125	16.76
Two years		220	29.49
Three years		245	32.84
Four years		79	10.59
More than four years		54	7.24
Does your husband/partner have another partner?			
No		637	85.39
Yes		109	14.61

**Table 3:** Aspects of household decision-making power in Jimma Zone, Western Ethiopia in 2020.

	Decision-Making Dimension (N = 746)	Joint or Alone Percent	Husband or Someone Else (%)
**1**	In your household who usually makes decisions about large household purchases	68.23	31.77
**2**	In your household who usually decides to visit your family, relatives	24.26	75.74
**3**	In your household who usually makes decisions about the health care of the women	19.84	80.16

**Table 4:** Respondent’s attitude towards physical violence towards wives in Jimma Zone, Western Ethiopia, 2020.

	Indicator	Yes (%)	No (%)	Total
	In your opinion, is a husband justified in physical violence towards his wife in the following situations:			
1	If she goes out without telling him?	66.62	33.38	100
2	If she neglects the children?	58.71	41.29	100
3	If she argues with him?	52.55	47.45	100
4	If she refuses to have sex with him?	40.21	59.79	100
5	If she burns the food?	45.84	54.16	100

**Table 5:** Factor analysis for indicators of empowerment (n = 746), Jimma Zone, Western Ethiopia, 2020.

Latent Construct	Aspects Asked about	Factor Loadings (EFA)	*p* Value (CFA)
Decision-making power	Decisions on big household purchases	0.244	
	Decisions on own health care	0.591	0.000
	Decisions on going to public meetings	0.635	0.000
Attitude towards physical violence	Physical violence if she neglects children	0.825	
	Physical violence if she argues with her partner	0.755	0.000
	Physical violence if she burns food	0.695	0.000
	Physical violence if she goes out without telling her partner	0.745	0.000
	Physical violence if she refuses to have sex	0.825	0.000

In Table 5, the path of the first indicator is constrained to 1, thus the significance value or *p* value was not calculated. Other variable factor loadings are significant at *p* < 0.05. The model’s fit was calculated by comparative fit index (CFI = 0.945), root mean square error approximation (RMSEA = 0.080), standardised root mean square residual (SRMR = 0.043).

**Table 6:** Standardised path coefficients of the latent variable SEM (n = 746), Western Ethiopia, 2020.

Predictors in the Equation (X):	Dependent Variables		
	Attitude Towards Violence	Decision-Making Power	Use of Family Planning
Endogenous variables			
1. Decision-making power			0.101 *
2.Attitude towards violence			0.104 **
Exogenous variables			
Current age of the women	0.089 *	0.463 ***	
Having access to information			0.192 ***
Having access to a health facility			0.140 ***
Having awareness about contraceptives	−0.118 **		0.240 ***
Husband being employed			0.091 **
Age of the husband		−0.338 **	−0.126 *
Being from middle and higher wealth class			−0.114 ***
Rural residence			−0.085 **
Occupation/no job or farmer			
Religion			
If she ever faced any form of violence	−0.116 **		
If she or husband has no habit of alcohol consumption	0.153 ***		
Partner not having habit of chewing/drinking	0.195 **	0.262 ***	
Has exposure to education	0.200 ***		
Source of income		0.153 **	
Husband being literate		0.223 **	

*** *p* < 0.001, ** *p* < 0.01, * *p* < 0.05.

## Supplementary Materials

The following are available online at https://www.mdpi.com/article/10.3390/ijerph18126550/s1, The raw dataset was uploaded as part of the Supplementary Materials under Supplementary File S1 and Supplementary File S2: Research questionnaire..

## Abbreviations

This paper has the following abbreviations: SEM: Structural equation modelling, SSA: Sub-Saharan Africa, SDG: Sustainable Development Goal, HIV: Human immunodeficiency virus, DHS: Demographic and health survey, DE: Design effect, TV: Television, SD: Standard deviation; CFA: Confirmatory factor analysis, RMSEA: Root mean square error of approximation, CFI: Comparative fit index and SRMR: Standardised root mean square residual.
