# Supplementary material for: The Effect of Women’s Empowerment in the Utilisation of Family Planning in Western Ethiopia: A Structural Equation Modelling Approach
Source: Int J Environ Res Public Health. 2021 Jun 18;18(12):6550. doi: 10.3390/ijerph18126550 (PMC8296516; doi:10.3390/ijerph18126550)
Supplement: Supplementary file 1 [file ijerph-18-06550-s001.zip › Supplementary Files 2 Questionaire.pdf]

QUESTIONNAIRE FOR MARRIED WOMEN

Dear Respondent

My name is (***your name***). We are from PBT Africa consultancy firm hired by AMREF Health Africa to conduct operation research on the effect of women empowerment on family planning service utilization in the project intervention areas. I would like to talk to you about your health and other topics. This interview will take about ***30 – 4o*** minutes. All the information we obtain will remain strictly confidential and anonymous. If you wish not to answer a question or wish to stop the interview, please let me know.

**May I start now?**<br>**Yes....................................1**<br>**No.......................................2**	2⇨*End*
**Identification**	**WM**
**Interviewer's name:**	**Date of data Collection**<br>*Day / Month / Year of interview:*<br>___ ___ /___ ___ / ___ ______ ___
**Zone**	1. Jimma Zone:<br>2. East wollega Zone:
**Woreda**	1. Dedo<br>2. Seka Chekoresa<br>3. Leka Dolecha<br>4. LekaDega
**Name kebele**	______________________
**Place of residence**	1. Urban<br>2. RURAL
**Geo location**	GPS reading

BACKGROUND		
**B1**. How old are you (age in completed years)?	Date of birth (Full Years) ________________	

**B2**. What is the highest level and grade or year of school you have attended?	1. Illiterate<br>2. Primary (1-8)<br>3. Lower Secondary (9-10)<br>4. Upper Secondary/Preparatory (11-12)<br>5. Technical/Vocational<br>6. Higher	
**B4**. What is your religion?	1. Christian Ortodox<br>2. Christian Protestant<br>3. Muslim<br>4. Catholic<br>5. Traditional<br>6. Non-believer	
**B5.** What is your current employment status	1. Working<br>2. Not working	
**B6.** What is your current occupation?	1. Student<br>2. Student with job<br>3. Employed and salaried<br>4. Trader- own business<br>5. Farmer<br>6. Daily labourer<br>7. No job at all	
**B6.** What is the main source of income of your family?	1. land cultivation<br>2. husbandry<br>3. paid job<br>4. labour<br>5. small business<br>6. remittance<br>7. Other________________________________	
**Perceived wealth status of the women**	1. Highest wealth<br>2. Wealthy<br>3. Middle class<br>4. Poor<br>5. Poorest	
**B7.** Are you currently married or living together with someone as if married?	1. Yes, currently married<br>2. Yes, living with a partner<br>3. Single	

**B8.** At what age did you marry with your first partner/husband	______________________	
**B9.** Do you drink alcohol (Tela, Arekie or chew any substance (Chat or cocokin) frequently	1. Yes<br>2. No	
**B10.** Access to source of information (TV/Radio/magazine)	1. Yes<br>2. No	
**B11.** How frequently do you listen to Radio/television or read news	1. Most frequently<br>2. Sometime<br>3. Rarely	
**Husband/partner Variables**		
**B12.** What is Age of your husband in full years	_____________	
**B13.** what was the highest level of school your husband attended?	1. Illiterate<br>2. Primary (1-8)<br>3. Lower Secondary (9-10)<br>4. Upper Secondary/Preparatory (11-12)<br>5. Technical/Vocational<br>6. Higher	
**B14.** Employment status of your husband in the past 12 months	1. Working<br>2. Not working	
**B15.** Does hedrink alcohol (Tela, Arekie or Chew any substance (Chat or cocokin) frequently	1. Yes<br>2. N0	
**FERTILITY/BIRTH HISTORY**		

**F1**. Now I would like to ask about all the births you have had during your life.<br><br>Have you ever given birth?	1. yes<br>2. No	
**F2.** How many children do you have in your life (both alive and dead)?	______________	
**F3**. Total number of children alive	Male_________<br>Female ______	
**F4. W**hat is the age of the young child (in full years)	____________	
F5. IF THE NUMBER OF CHIDREN AREMORE THAN TWO A WOMAN HAS, WHATIS THE AGE DIFFERENCE BETWEEN THE YOUNGEST AND HIS/HER IMMEDIATE ELDER BORTHER/SISSTER (SPACING BETWEEN BIRTH)	1. One year<br>2. Two year<br>3. Three year<br>4. Four year<br>5. Above that	
DOES YOUR HUBAND HAVE ANOTHER PARTNER OTHER THAN YOU	1. YES<br>2. No	

**Contraception**		
I would like to talk with you about family planning.<br><br>**CP1**. Are you pregnant now?	1. Yes, Currently Pregnant<br>2. No	
Have you ever heard about contraceptive to prevent pregnancy	Yes<br>No	If no skip to xx

**CP2**. Please name the methods that you know about to delay or avoid pregnancy<br><br>*Do not prompt.*<br>*If more than one method is mentioned, record each one.*	1. Female sterilization<br>2. Male sterilization<br>3. IUD<br>4. Injectables<br>5. Implants<br>6. Pill<br>7. Male condom<br>8. Female condom<br>9. Diaphragm<br>10. Foam / Jelly<br>11. Lactational amenorrhoea method (LAM)<br>12. Periodic abstinence / Rhythm<br>13. Withdrawal<br>14. Other (specify)____________________	
**CP3**. Do you support the use of contraceptive to prevent pregnancy	1. Yes<br>2. No	
**CP4**. Have you ever done something or used any modern contraceptive method to delay or avoid getting pregnant?	1. Yes<br>2. No	
**CP5**. If you are not pregnant , Are you currently doing something or using any method to delay or avoid getting pregnant?	1. Yes<br>2. No	If no skip FP1
**CP6.** what are you doing to delay or avoid a pregnancy currently?<br><br>*Do not prompt.*<br>If more than one method is mentioned, record each one.	1. Female sterilization<br>2. Male sterilization<br>3. IUD<br>4. Injectables<br>5. Implants<br>6. Pill<br>7. Male condom<br>8. Female condom<br>9. Diaphragm<br>10. Foam / Jelly<br>11. Lactational amenorrhoea method (LAM)<br>12. Periodic abstinence / Rhythm<br>13. Withdrawal<br>14. Other (specify)____________________	

ACCESS AND USE OF FP SERVICES		
**FP1.** Do you have access to health facility in nearby (Health centre/health post)	1. Yes<br>2. No	
**FP2.** Have you ever visited a health facility or doctor to receive services or information?	1. Yes<br>2. No	
**FP3.** What is your closets facility that you go normally	1. Health post<br>2. Health center<br>3. Hospital<br>4. Traditional healer	
**FP4.** How far is the distance from the health facility to your home?	1. Less than 10 Km<br>2. Greater than 10km	
**FP5.** How long does it take for you? In hours	1. Less than 2hours<br>2. More than 2 hours	
**FP6.** Do you have access to transport to go to health facility whenever you need	1. Yes<br>2. No	
**FP7.** Does the cost of transport and/or treatment prevent you to got to health facility	1. Yes<br>2. No	
**FP8.** Are you happy on the quality of service provided by the closest health facility	1. Yes<br>2. No	
**FP9.** Do you think the health facility have enough health care providers to treat or give the service you need	1. Yes<br>2. No	

Decision Making Behaviour Related to Household Economy and Socio-Cultural Activities		
Let´s now talk about decision making within the households,<br><br>**DM1.** In your household who usually makes decisions about large household purchases?<br><br>*Probe: (give local examples of large purchases)*	1. Mainly You<br>2. Mainly Your husband<br>3. Joint Decision (Both you & your husband)<br>4. Someone else In-law	
**DM2.** In your household who usually makes decisions about household purchases for daily needs?	1. Mainly You<br>2. Mainly Your husband<br>3. Joint Decision (Both you & your husband)<br>4. Someone else	
**DM3.** In your household who usually decides how to use money that *you* bring into the household?	1. Mainly You<br>2. Mainly Your husband<br>3. Joint Decision (Both you & your husband)<br>4. Someone else	
**DM4.** Would you say that the money that you earn is more than what your husband/partner earns, less than what he earns, or about the same?	1. less than what he earns<br>2. the same<br>3. more than what he earns	
**DM5.** Who usually decides how your husband's earnings will be used?	1. Mainly You<br>2. Mainly Your husband<br>3. Joint Decision (Both you & your husband)<br>4. someone else	
**DM6.** In your household who usually decides when your family will sell a small asset (like a chicken)?	1. Mainly You<br>2. Mainly Your husband<br>3. Joint Decision (Both you & your husband)<br>4. someone else	
**DM7.** In your household who usually decides to visit to your family, relatives or public spaces? Probe like health facility, Market, Public meeting etc.	1. Mainly You<br>2. Mainly Your husband<br>3. Joint Decision (Both you & your husband)<br>4. someone else	

**DM8**. In your household who usually decides About the size of the family (how many children to have…)	1. Mainly You<br>2. Mainly Your husband<br>3. Joint Decision (Both you & your husband)<br>4. someone else<br>5. it is only in the hands of god	
**Health seeking behavior**		
**HB1.** In your household who usually makes decisions about the health care of the women?	1. Mainly You<br>2. Mainly Your husband<br>3. Joint Decision (Both you & your husband)<br>4. Someone else	
**HB2.** In your household who usually makes decisions about the use of contraceptives?	1. Mainly You<br>2. Mainly Your husband<br>3. Joint Decision (Both you & your husband)<br>4. someone else	
**HB3.** If your child is sick /feels sick, do you go to health facility	1. Yes<br>2. No	
**HB4.** If yes how soon are you to take a sick child to health facility	1. Immediately in one days<br>2. Waits two to three days<br>3. May take the child after a week if he is not cured	
**HB5.** If no, what is the reason for not taking your child to health facility	1. I feel my child will cure by its own<br>2. Because I afraid my husband<br>3. I have no money<br>4. The facility is far<br>5. I have no trust the health facility	

Women Attitude towards wife beating

Sometimes a husband is annoyed or angered by things that his wife does. In your opinion, is a husband justified in hitting or beating his wife in the following situations:

WDV 1. If she goes out without telling him?	1. Yes<br>2. No	
WDV 2. If she neglects the children?	1. Yes<br>2. No	

WDV 3. If she argues with him?	1. Yes<br>2. No	
WDV 4. If she refuses to have sex with him?	1. Yes<br>2. No	
WDV 5. If she burns the food?	1. Yes<br>2. No	
Sometimes a husband is annoyed or angered by things that his wife does. In your husband opinion, is a husband justified in hitting or beating his wife in the following situations:		
MDV 1. If she goes out without telling him?	1. Yes<br>2. No	
MDV 2. If she neglects the children?	3. Yes<br>4. No	
MDV 3. If she argues with him?	1. Yes<br>2. No	
MDV 4. If she refuses to have sex with him?	1. Yes<br>2. No	
MDV 5. If she burns the food?	1. Yes<br>2. No	
Does your husband feels that you cheating him	3. Yes<br>4. No	
Does your husband jealous and limit you not to meet your relatives and friends	5. Yes<br>6. No	
**WDV 6.** Have you ever experienced one the following by your partners push, shook, threw something, twisted, punched)	1. Yes<br>2. No	
**WDV 7.** Have you ever experienced emotional violence (threatened, make you feel bad, threatened to harm)	1. Yes<br>2. No	

**WDV 8.** Have you ever experienced sexual violence (forcefully or without consent t)	1. Yes<br>2. No	
**Interviewer's Observations**		
